# Accuracy of digital workflow for placing orthodontic miniscrews using generic and licensed open systems. A 3d imaging analysis of non-native .stl files for guided protocols

**DOI:** 10.1186/s12903-023-03113-9

**Published:** 2023-07-17

**Authors:** Vincenzo Ronsivalle, Pietro Venezia, Orazio Bennici, Vincenzo D’Antò, Rosalia Leonardi, Antonino Lo Giudice

**Affiliations:** 1 Department of General Surgery and Medical-Surgical Specialties, Via Santa Sofia 78, University of Catania, Catania, Italy; 2 Orthodontic Graduate Program, University of Federico II, Naples, Italy

**Keywords:** Digital dentistry, Digital orthodontics, Digital planning skeletal anchorage, TADs

## Abstract

**Background:**

This study aimed to assess the accuracy of digital workflow for guided insertion of miniscrews in the anterior palate using restorative implant dentistry software and licensed software for orthodontic applications.

**Methods:**

Twenty subjects (8 males, 12 females, mean age = 16.7 ± 2.1 years) were prospectively selected to receive guided insertion of bicortical palatal miniscrews. Virtual planning was performed using restorative implant dentistry software (Blue Sky Plan*, version 4.7) (group 1 = 10 subjects) and licensed orthodontic software (Dolphin Imaging Software, version 11.0) (group 2 = 10 subjects). A specific 3D Imaging technology was applied to permit the registration of the planned and achieved position of the miniscrews based on the superimposition of maxillary models. The angular deviation (accuracy error) between the planned and the achieved positions of the miniscrews were recorded. Independent Student’s test was used with statistical significance set at p value < 0.05.

**Results:**

The mean accuracy error recorded in group 1 was 7.15° ± 1.09 (right side) and 6.19 ± 0.80 (left side) while the mean error in group 2 was 6.74° ± 1.23 (right side) and 5.79 ± 0.95 (left side). No significant differences were recorded between the two groups (p > 0.05); instead, miniscrews placed on the right side were almost one degree higher than the left side (p < 0.05) in both groups.

**Conclusions:**

The clinical accuracy error was similar when using generic and licensed orthodontic software for guided systems.

**Supplementary Information:**

The online version contains supplementary material available at 10.1186/s12903-023-03113-9.

## Introduction

Skeletal anchorage is used to simplify complex orthodontic biomechanics, particularly when patient compliance and lack of residual skeletal growth can affect treatment outcomes [1]. Among extra-alveolar areas for miniscrews insertion, anterior palatal bone and infra zygomatic crest showed a higher success rate compared to the mandibular retromolar area and the mandibular buccal shelf [2–5]. The maxillary paramedian region is considered an excellent area for miniscrews insertion because it offers adequate bone depth and two cortical laminae for miniscrews stability [4, 6–8]. Also, it is a safe zone due to the absence of sensitive anatomical structures [9, 10]. The anterior palate is a generous area, even from the biomechanical perspective, since it offers anchorage for orthodontic or orthopedic forces applied in the sagittal, transverse, and vertical directions.

Although free-hand insertion of miniscrews in the anterior palatal is a safe procedure [11], the literature suggests to use a digital guided system for two reasons: first, to better control miniscrews inclination and parallelism, which facilitates orthodontic appliance placement and prevents bone trauma during insertion [12, 13]; second, to plan more precisely the relationship between the miniscrews and the cortical palatal and nasal bone [14].

Guided miniscrews insertion can be planned with different software available in the market, using a lateral cephalogram or a cone-beam computed tomography (CBCT) registered with a digital intraoral scan. To avoid unnecessary radiation exposure to patients, CBCT should be used when in-depth analysis of specific anatomical conditions is required [15]. Both methods are accurate, despite the guided clinical insertion is not exempt from some degree of error [11, 16, 17]. Generally, the software used to plan the insertion of miniscrews (including those used in previous studies) are explicitly designed for orthodontic applications, can be integrated with laboratory software, or are provided by companies to be used strictly with their miniscrews. The advantage of these systems is the efficiency of the workflow, from the planning stage to the appliance delivery (including full-digital CAD-CAM applications). At the same time, the disadvantage is the high cost of the software (purchase or yearly subscription) or the single case (planning/appliance fabrication package).

Open systems designed for implant dentistry are also available and can be used for planning the insertion of orthodontic minscrews and designing surgical guides. Limitations are the inability to use native .stl files of the miniscrew/scan-bodies and lateral cephalogram for preliminary registration. The advantages are the versatility of the system and the reduced cost (limited to the printing of surgical guides) [12]. For these reasons, the open systems represent a valid alternative for the digital-native generation of clinicians who are at the beginning of their clinical experience and are not supported by solid financial sustainability.

Nevertheless, there is no evidence in the literature concerning the accuracy of open systems for planning guided insertion of orthodontic miniscrews. In this regard, the present study aimed to assess the accuracy of the workflow for guided insertion of miniscrews in the anterior palate using two open systems, one restorative implant dentistry software and one well-known licensed software designed for orthodontic applications. The null hypothesis was the absence of significant differences in the angular deviation between the planned and the final achieved position of miniscrews using different software.

## Methods

The present study included twenty subjects (8 males, 12 females), with a mean age of 16.7 ± 2.1 years, consecutively treated by two clinicians between August 2019 and December 2022, and was approved by the Institutional Ethical Committee of the University of Catania (protocol n. 119/2020/PO). Inclusion criteria were: indication for anterior palatal skeletal anchorage, indication for CBCT-based miniscrews guided insertion (impacted teeth, ectopic palatal position of the lateral incisor, narrowed palate), or for pre-surgical planning of upper third molars requiring CBCT scans. Exclusion criteria were: previous orthodontic treatment, systemic disease, cleft palate, and use of drugs influencing bone metabolism.

The digital workflow consisted of several steps for planning the insertion of miniscrews and evaluating the consistency between the planned and achieved position of the miniscrews.

### Preliminary measurements of the miniscrews and components for guided system

**Fig. 1:**
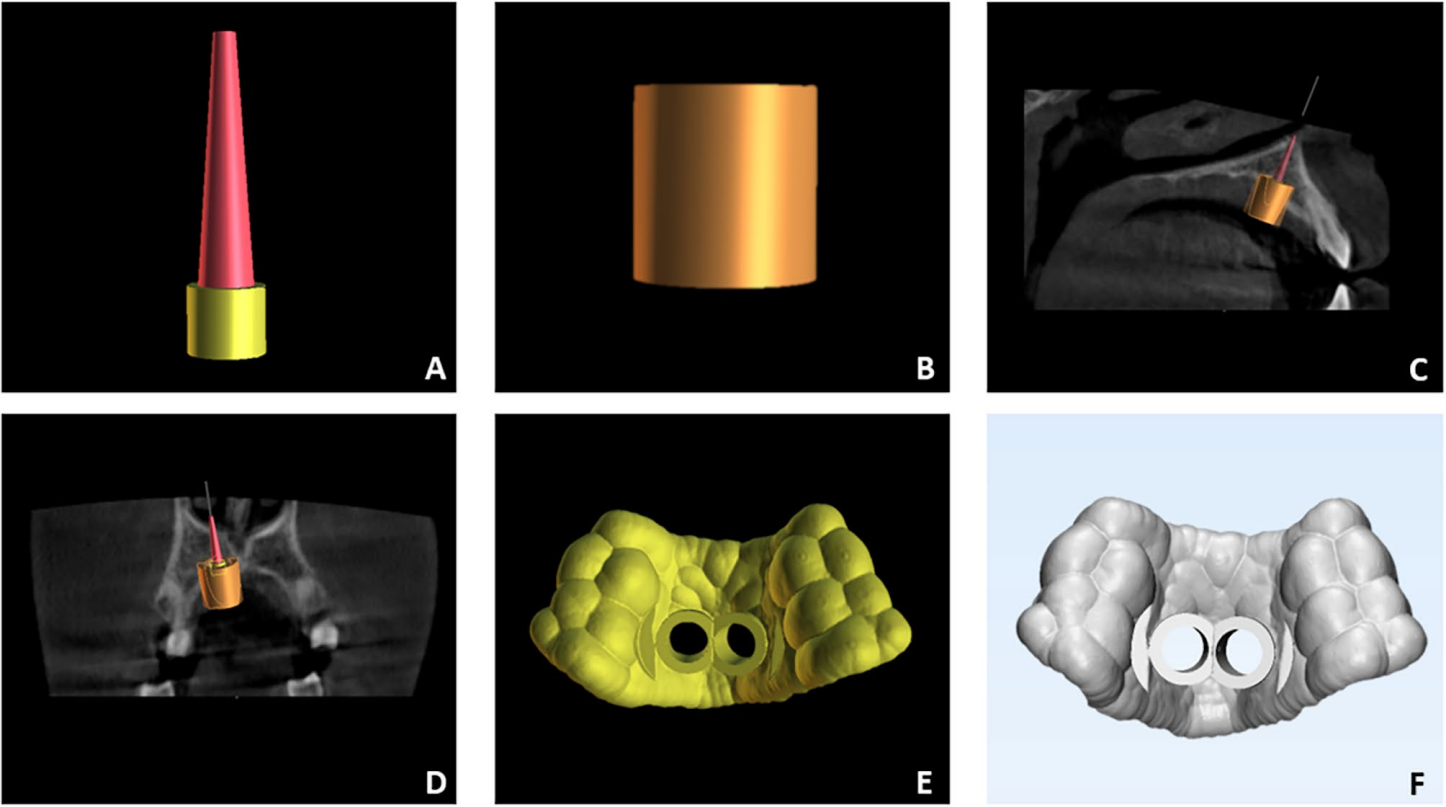
Iconographic representation of the digital work-flow used for planning miniscrews insertion using BlueSky Plan software and non-native .stl files. (**A**) customized virtual miniscrew; (**B**) customized virtual surgical tube according to the measurements of the pick-up driver; (**C**) sagittal view of bicortical anchorage planned; (**D**) coronal view of bicortical anchorage planned; (**E**) surgical guide designed; (**F**) surgical guide exported for the further steps of the analysis

All subjects received Spider Screws Regular Plus Konic (HDC Srl, Vicenza, Italy) 2 mm in diameter and 9, 11, or 13 mm in length according to the depth necessary to achieve bicorticalism. Since the study was performed without the native .stl file of the miniscrews, measurements of the miniscrews and of the components of guided insertion systems were preliminary calculated using a digital caliper [12]: miniscrews = screw body length, screw apical body diameter (apical diameter), screw occlusal body diameter (occlusal diameter), intra-oral head length, intra-oral head diameter; components for guided system = pickup diameter, pickup length (up to the occlusal stop), linear extension from the end of the miniscrew neck to the occlusal limit of the pickup length (for the definition of the offset of the surgical tubes). These measurements served to generate customized miniscrews and design surgical tubes (Fig. 1a,b).

### Digital planning

The intra-oral scan (IOS) and CBCT scans of each patient were imported into Blue Sky Plan* (Blue Sky Bio, version 4.7, Grayslake, IL, USA), a certified software typically employed in restorative implant dentistry. The software allows a preliminary point-based superimposition between IOS and CBCT, followed by final registration based on the best-fit algorithm. The “customize implant” function was used to create a virtual equivalent miniscrews with an abutment representing the extra-alveolar screw head, using the measurements registered in the preliminary step. The orthodontic miniscrew can be considered a monophasic temporary implant with an intraosseous portion, an intramucosal neck, and an intraoral portion (miniscrew head). The intraosseous portion and gingival neck are part of the screw body, while the head of the miniscrew is the abutment (Fig. 1a).

The first operator performed the virtual placement of the miniscrews in the paramedian area at the level of the third palatal ruga, adjusting the position and orientation of the screws in axial, coronal, and sagittal views as well as in the 3D rendering and assuring to reach adequate depth for bicorticalism (Fig. 1c-d). Afterward, the surgical tubes were designed according to the measurements of the pick-up driver (Fig. 1b) to ensure that the screws were inserted at the correct angle. Finally, the surgical guide was generated (Fig. 1e). The maxillary IOS and the surgical guide were exported as .stl files (Fig. 1f).

Before planning digital insertion with Blue Sky Plan software, the equivalent miniscrew was exported as .stl file and used with the second tested software. In this regard, another operator performed the virtual placement of the miniscrews with the Dolphin Imaging Software, (Dolphin Imaging, version 11.0, Chatsworth, CA, USA) (Supplementary Fig. 1), using the same customized miniscrews and the same indications for miniscrews insertion in the anterior palate, including bicorticalism. The surgical tubes and guide were designed, and the maxillary IOS and the surgical guide were exported as .stl files. Both operators were highly skilled orthodontists with more than 5 years of experience in guided systems and with the tested software.

All .stl files of surgical guides were sent to the same laboratory and prototyped with the same apparatus and printing settings: 3D Printer = Form 2 (Formlab, Somerville, MA, USA); resin = SG Resin (Formlab, Somerville, MA, USA); printing settings = 30° inclination from printing platform, layer thickness 0.50 μm; post-printing settings = 2 separate immersion baths of 97% isopropyl alcohol, air drying at room temperature for 30 s, curing process at wavelength 385–405 nm (Form Cure machine Formlab, Somerville, MA, USA).

### Miniscrews insertion pillars and cover body design

**Fig. 2:**
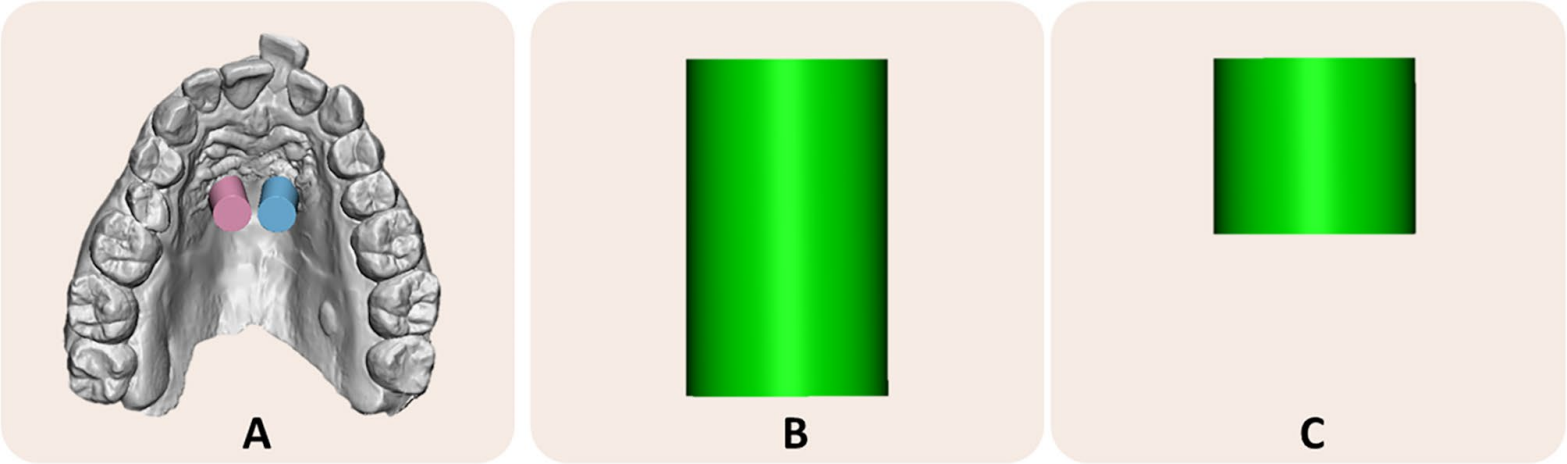
Cover Body designed to allow analysis of accuracy error in absence of original .stl file of the scanbody. **A**) Master model with pillars generated fulfilling the inner portion of digital surgical guide; **B**-**C**) cover body designed to host the original scanbody and generated from a new cylindrical pillar that was cut to exclude interferences with palatal mucosa

The .stl file of the IOS and surgical guide were imported into Shapr3D software (Shapr3D Zrt, Budapest, Hungary) to generate intra-oral pillars that indicated the orientation of the planned miniscrews’ position. For this purpose, two cylindrical pillars were generated by fulfilling the inner portion of the surgical tubes and were merged with the IOS (boolean function) to obtain the master model of the analysis (File A) (Fig. 2a). Afterward, a customized cover body was designed and prototyped to allow subsequent analysis between the planned and post-insertion positions of the miniscrews. In particular, the cover body was designed to host the original scanbody during the post-insertion IOS acquisition. The cover body was generated from a new cylindrical pillar that was cut to exclude interferences with palatal mucosa (Fig. 2b-c).

### Miniscrews insertion and IOS acquisition

**Fig. 3:**
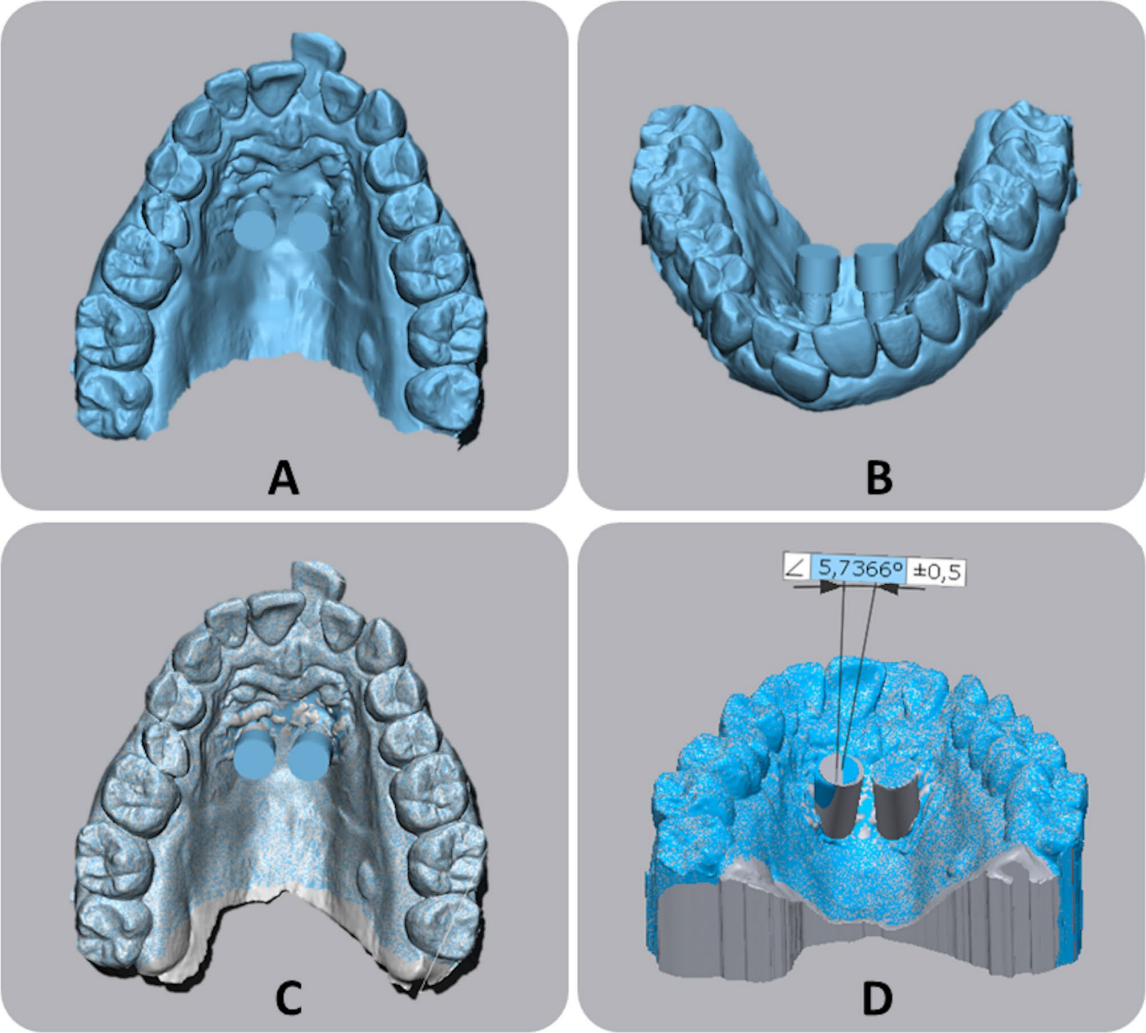
Work-flow for digital analysis of accuracy error of miniscrew insertion. **A**-**B**) Intra-oral scan with cover-bodies applied onto scan-bodies (File B); **C**) superimposition of master model (File A) with intra-oral scan; **D**) calculation of angular differences between pillars and cover bodies

After local anesthesia (2% lidocaine), the surgical guide was fitted to the occlusal surfaces of the dentition. Predrilling was performed using a calibrated bur. Two Spider Screws Regular Plus Konic miniscrews were inserted into the pick-up driver and mounted on a contra-angle handpiece at a low speed of 35 rpm. Polyetheretherketone (PEEK) scan bodies (HDC Srl, Vicenza, Italy) with the cover body were fixed on the screws to obtain IOS registration of the miniscrews’ position (File B) (Fig. 3a).

### Digital analysis

Files A and B were imported into Geomagic Control X software (3D Systems, version 2018.1.1, 3D Systems, USA) (Fig. 3b). Firstly, a point-based registration was performed using the distobuccal cusps of the upper first molars and the mesial angle of the incisal edges of the central incisors, followed by the final registration of the two models based on the automated best-fit algorithm (Fig. 3c).

The function “builded geometry” was used to recognize the cylindric geometry of both pillars (File A) and cover bodies (File B). Once cylinders were selected, the software automatically calculates the vector of both pillars and cover bodies (“add vector function”). Finally, the longitudinal axes were drawn, and the angular measurements between pillars and cover bodies were performed using the function “find axis of the cylinder” (Fig. 3d).

Digital analysis was performed by a single operator (V.R.), and the procedure was repeated two weeks later to analyze intra-observer variability and method error. A second operator (A.L.G.) also performed the procedure to assess reliability among observers. All data were collected and categorized on an Excel spreadsheet to perform statistical analysis.

### Statistics

Data for sample size calculation were retrieved from previous published data [16]. The power analysis found that a sample size of 6 subjects per group achieved 95% power to detect an average angular difference of 5.70° between planned and achieved miniscrews position, with a known standard deviation of 3.42 ° and with a significance level set at 0.05.

However, we decided to tested 10 models for each group, increasing the power of the available data.

Descriptive statistics were carried out to the angular differences between planned and achieved miniscrews insertion procedures. Shapiro-Wilk and Levene’s tests were used to calculate normal distribution and equality of data variance. Since all data showed normal distribution and equality of the variance, parametric tests were used for analyzing data outcomes. A preliminary comparison of the angular measurements recorded between the right and left sides of the palate was performed using paired Student’s test. The Independent Student test was used to compare the angular differences obtained with different software. The intraclass correlation coefficient (ICC; model = 2-way mixed effects, type = single measure, definition = absolute agreement) was performed to calculate intra-examiner and inter-examiner reliability of the superimposition and measurement workflow. Data were analyzed using SPSS® version 24 Statistics software (IBM Corporation, 1 New Orchard Road, Armonk, New York, USA).

## Results

**Fig. 4:**
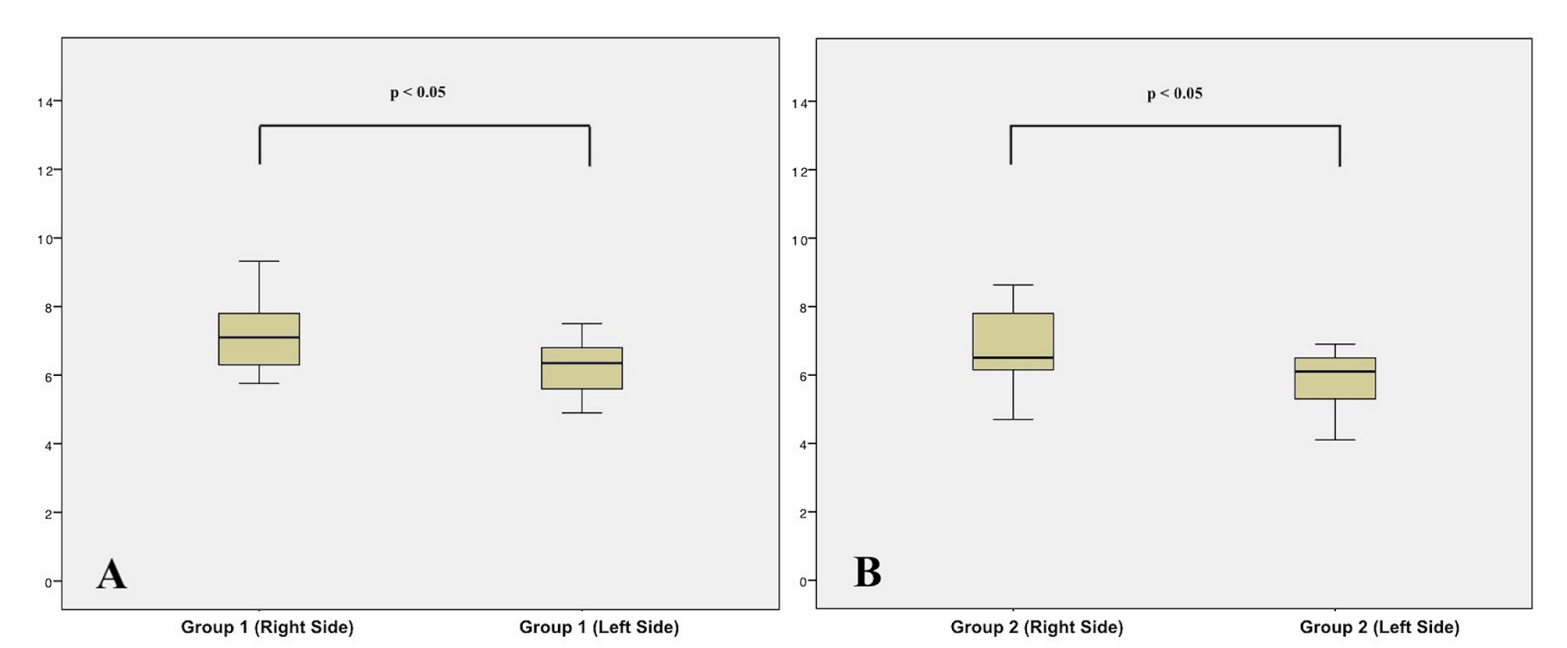
Boxplots of the distribution of clinical accuracy error and significance between right and left side in both group 1 (**A**) and group 2 (**B**)

**Fig. 5:**
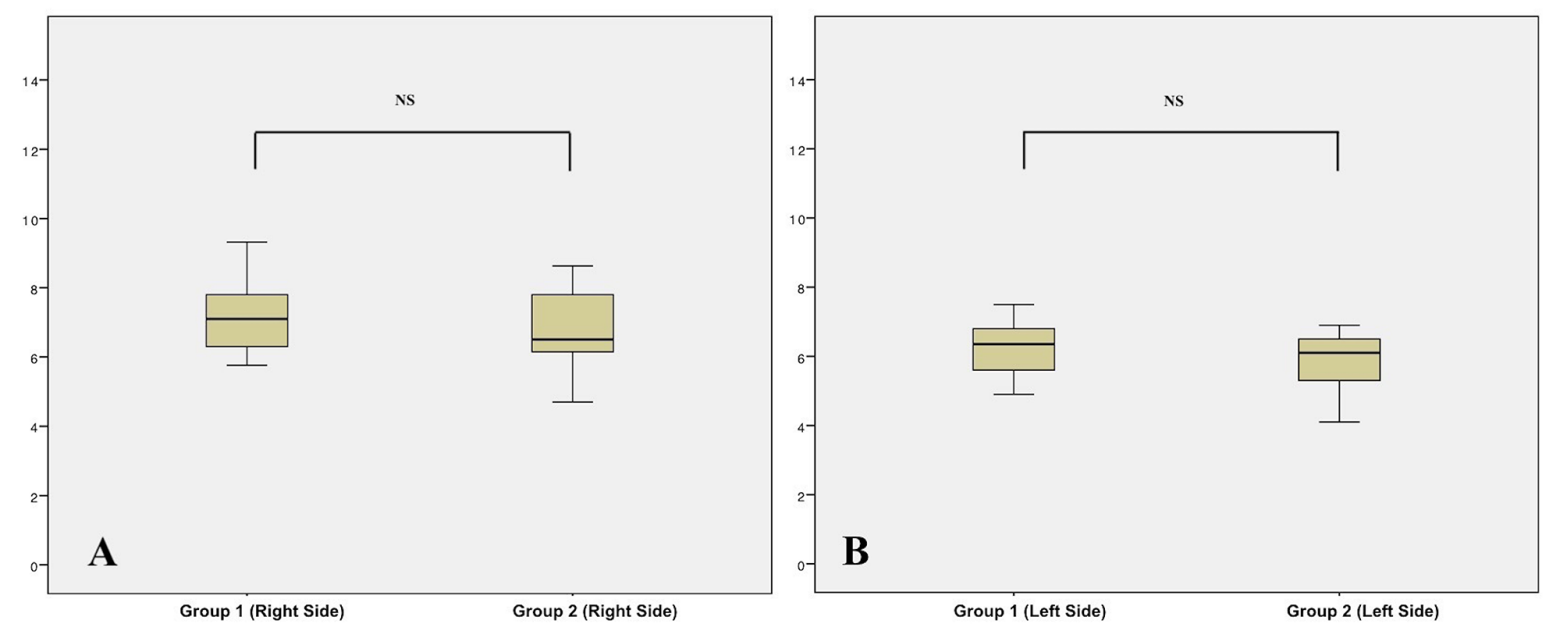
Boxplots of the distribution of clinical accuracy error and significance between group 1 and group 2 separating right side (**A**) and left side (**B**)

Table 1 shows descriptive statistics of the angular differences between planned and achieved miniscrews insertion. Since significant differences were found between the right and left sides, tables reported data outcomes distinguishing angular measurements from both sides (Tables 1 and 2). In particular, the mean error of miniscrews placed on the right side was almost one degree higher than the left side (p < 0.05) in both groups (Table 2; Fig. 4). The mean error recorded in group 1 (Blue Sky Plan software) was 7.15° ± 1.09 (right side) and 6.19 ± 0.80 (left side); the mean error in group 2 (Dolphin software) was 6.74° ± 1.23 (right side) and 5.79 ± 0.95 (left side). No significant differences were recorded between the mean error generated in groups 1 and 2 (p > 0.05) (Table 2; Fig. 5). Concerning the reliability of the measurements, ICC tests showed no difference between the two readings with an excellent correlation ranging from 0.879 to 0.920 for intra-observer reliability and 0.852 to 0.887 for inter-observer reliability.

**Table 1:** Descriptive statistics of the angular differences between planned and achievde miniscrews insertion

	Group 1			Group 2			
	Right Side	Left side		Right Side	Left side		
Mean	7.15	6.19		6.74	5.79		
SD	1.09	0.80		1.23	0.95		
SE	0.34	0.25		0.41	0.30		
Min	5.76	4.90		4.70	3.90		
Max	9.32	7.50		8.63	6.90		

Group 1 = BlueskyPlan Software; Group 2 = Dolphin Software

SD = standard deviation; SE = standard error; Min = minimum value; Max = maximum value

**Table 2:** Inferential statistics of the angular differences between planned and achieved miniscrews insertion

Groups	Side	N	Mean	SD	Significance	Mean Diff.	SE Diff.	95% Interval Coefficient	
								Lower Limit	Upper Limit
Group 1	Right	10	7.15	1.09	0.44*	0.41	0.52	-0.68	1.50
Group 2		10	6.74	1.23					
Group 1	Left	10	6.19	0.80	0.32*	0.40	0.39	-0.43	1.23
Group 2		10	5.79	0.95					
Group 1	Right	10	7.15	1.09	0.002**	0.962	0.427	0.06414	1.85986
	Left	10	6.19	0.80					
Group 2	Right	10	6.74	1.23	0.019**	0.954	0.491	0.0783	1.98639
	Left	10	5.79	0.95					

*P value set at p < 0.05 and based on Independent Student t test. P < 0.05 = statistically significant

**P value set at p < 0.05 and based on paired Student t test. P < 0.05 = statistically significant

Group 1 = BluSkyPlan software; Group 2 = Dolphin software; N = number; SD = Standard Deviation;

Mean Diff. = difference between means; SE diff. = standard error of the difference

## Discussion

Previous evidence suggests that digitally guided systems permit to insert orthodontic miniscrews accurately and more precisely than the direct method [7, 18]. From the clinical perspective, it is essential to assess the difference between the predicted and achieved position of the miniscrew and the potential source of error using specific workflow for guided systems. In this regard, this is the first study in the literature that investigates the accuracy of miniscrews insertion in the anterior palate region using restorative implant dentistry software comparing data outcomes with those obtained using licensed planning software designed for orthodontic applications. For this purpose, we generated a cover body that allowed the surface-matching procedure without the original digital scan-body .stl file. The method used has shown excellent intra-operator and inter-operator reliability and could be used in future studies for analyzing a more comprehensive sample size or to assess the accuracy error of different types of miniscrews when the native .stl file of the miniscrew is not available.

In the present study, the clinical error generated between planned and placed mini-screws ranged from 5.79° to 7.15° on average. Our findings are similar to those reported in previous studies testing respectively monocorticalism [17] and bicorticalism [14, 16] bone anchorage. However, in two studies, the authors used analogic surgical guides made of two-components silicone [17] or thermoformed polyethylene terephthalate glycol [14]. Both materials may allow some drill sleeve mobility during pick-up descent, compared to the rigidity of printable resin used in the present investigation [17]. 3D printed surgical guides can also introduce some bias related to the trueness error in the printing workflow. In this regard, there is an urgent need for comparative studies assessing the accuracy error between analogic and 3D-printed surgical guides. Cassetta et al. [19] used a 3D-printed surgical guide and found an average error of 4.60°, which is remarkably below the error found in the present study. However, the study mentioned above reported a small sample size (5 subjects), and the wide range of standard deviation found by the authors makes the data difficult to compare.

The present findings can also be interpreted in terms of inter-operator comparisons. In this regard, the assumption was that the diameter of the metal sleeve (wider than the pick-up to allow frictionless insertion) could leave some degree of freedom introducing manual error during miniscrews insertion. Although a well-codified guiding system is not exempt from clinical error, the analogous accuracy error found between the two operators and the similarity of our findings with those available in the literature suggest that such inaccuracy is kept within a limited range. Nevertheless, a clinical error can be influenced by different variables ascribable to both patient and clinician [20]. In particular, bone quality and clinical expertise can have a significant rule, especially when both the palatal and the lower nasal cortical bone needs to be perforated [21].

Surprisingly, the miniscrews placed on the right side showed a slight greater accuracy error (about 1 degree) than those placed on the left side, reaching statistical significance in one of the two operators. This data might be explained considering that both operators were handed-right and it may be assumed that both operators may have introduced some adaptive twisting wrist movements during the pick-up descent on the right side to compensate for the deficient direct view of the surgical tube/pick-up components. However, this finding is in contrast with previous studies where no differences were found between right and left side, [14, 16, 17].

In light of the available literature and considering the present findings, a certain degree of inaccuracy exists even if we use static guided systems. In general, the accuracy error is limited to a few degrees and can be considered clinically acceptable since no dangerous structures are present in the paramedian region. However, the accuracy error can be clinically relevant when it influences the concordance between the lab procedure and the clinical insertion, especially in the one-visit protocol [22]. In this regard, Migliorati et al. [14] suggested that the angular deviation of miniscrew becomes clinically relevant when it affects the tolerance of the appliance system (device/miniscrew and teeth in case of hybrid anchorage), i.e., with the increased number of miniscrews, undercuts and rigidity of the appliance. In those circumstances, the dual-visit procotol should be preferred. The present study did not include the analysis of the accuracy of the laboratory step since the .stl model exported for the lab technician (a model with empty holes for the analogs) may have introduced some bias during the analog insertion due to the absence of the exact mathematics of the miniscrews. For the same reason, the application of BluSkyPlan software is actually limited to the production of the surgical guide and dual-visit appliance delivery protocol.

The accuracy error generated in the guided work-flow was similar between the two open systems, i.e., the generic restorative implant dentistry software (BluSkyBio) and the orthodontic licensed software (Dolphin). This finding would suggest that generic implant dentistry software is adequate for planning miniscrew insertion as licensed and more expensive orthodontic software. Open-source software represent another possibility for planning the insertion of orthodontic miniscrews, however there is only one study in the literature addressing this topic, and it was designed for prosthetic implant surgery [23].

Some clinical considerations should be addressed and are related to the usability of open systems for digitally guided miniscrews insertion systems. While companies offer digital systems and platforms for guided insertion of their miniscrews, open systems software has some advantages. Firstly, an open system is adaptable to all available miniscrews in the market, which streamline the workflow for those clinicians who use different miniscrews systems. Secondly, open-source software reduces cost and can be easily integrated with in-office production or improve communication between the clinician and the laboratory, who can share multiple projects using cloud-based files on the same software platform.

Actually, there are also disadvantages using open systems. The first limitation is that BSB does not allow the registration of the intra-oral scan with L-L radiograph, which limits the usability of the software only for those cases where CBCT acquisition is justified [15]. The second limitation is that the absence of a native digital file of miniscrews in the library does not allow to plan a fully-digital CAD-CAM workflow for appliance fabrication. However, a stepwise transition from analogic orthodontics to digital orthodontics has been recently suggested by Graf et al., even considering financial sustainability. In this regard, slow adaptation to digital systems is encouraged until cost and efficiency allow for complete digitalized orthodontic applications [24].

### Limitations

- Although the data recorded were consistent between both groups, the small sample size still represent a major concern of the present investigation.
- The present study did not provide information about the angular or linear deviation involving the intra-osseous portion of the miniscrews and is limited to the analysis of the inclination of the extra-mucosal head. From the clinical perspective, this should not be considered a major concern since the palatal paramedian region is a safe zone and the potential discrepancy between planned and achieved position of the miniscrew’s apex should not interfere with the integrity of sensitive structures (naso-palatine nerve).

## Conclusion

The following conclusions can be drawn according to the present findings:

- A certain amount of clinical deviation can occur between planned and achieved position of orthodontic miniscrews in the anterior palate.
- The amount of clinical accuracy error is similar when the digital work-flow is performed with restorative implant dentistry software and licensed orthodontic software.
- The operator’ dominant hand may represent a clinical variable that may influence the accuracy of guided miniscrews insertion between palatal opposite sides.

## Electronic supplementary material

Below is the link to the electronic supplementary material.

Supplementary Material 1

Supplementary Material 2

## Data Availability

The datasets used and analysed during the current study are available from the corresponding author on reasonable request.

## Abbreviations

CBCT: Cone-beam computed tomography
CAD-CAM: Computer aided design - Computer aided manufacturing
IOS: Intra-oral scan
3D: Three-dimensional
PEEK: Polyetheretherketone
L-L: Latero-lateral
