# Supplementary figures and images for: Accuracy of digital workflow for placing orthodontic miniscrews using generic and licensed open systems. A 3d imaging analysis of non-native .stl files for guided protocols
Source: BMC Oral Health. 2023 Jul 17;23:494. doi: 10.1186/s12903-023-03113-9 (PMC10353103; doi:10.1186/s12903-023-03113-9)

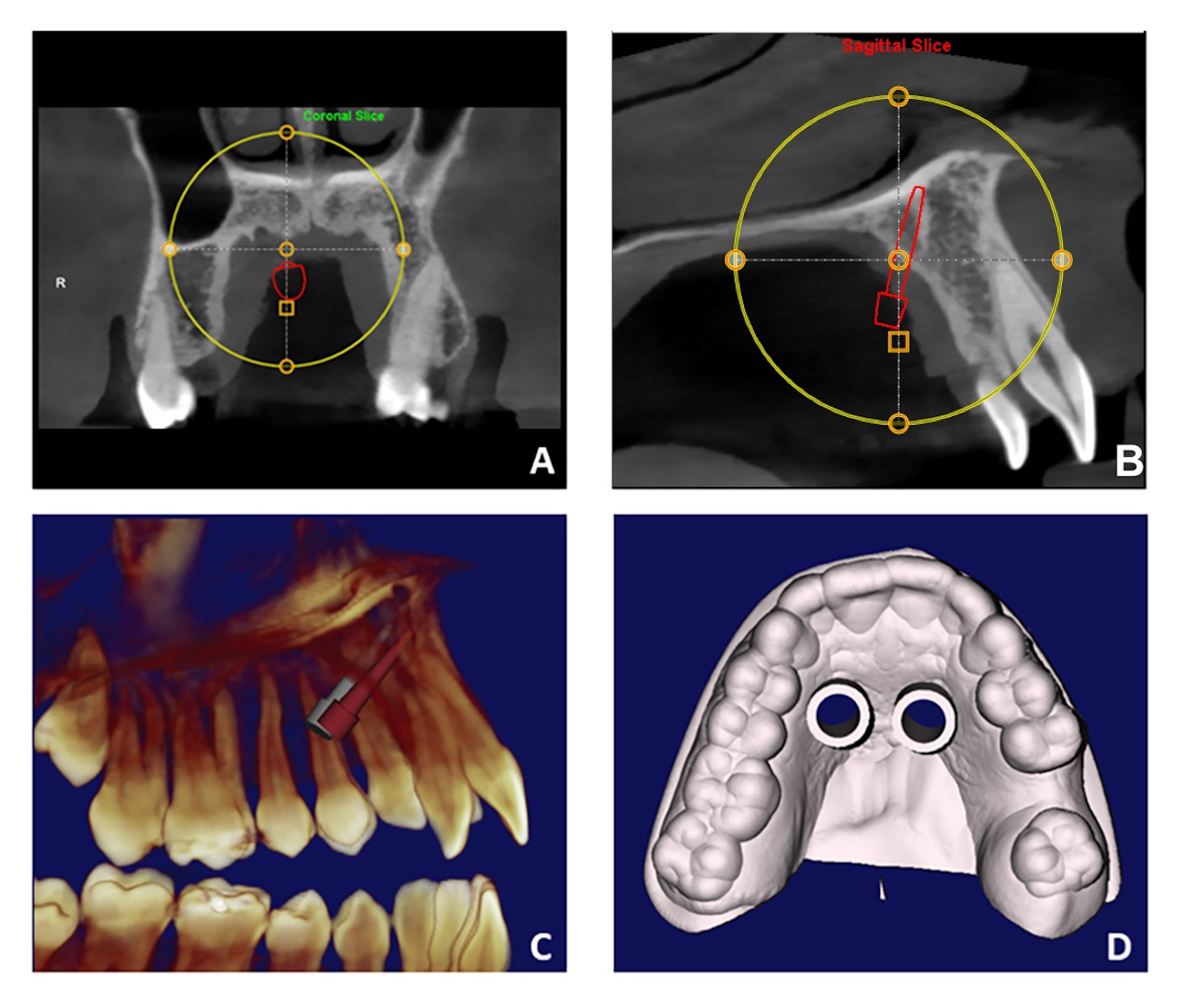

Supplement: Supplementary file 1 — Supplementary Material 1 [file 12903_2023_3113_MOESM1_ESM.png]
