# Supplementary material for: Accuracy of digital workflow for placing orthodontic miniscrews using generic and licensed open systems. A 3d imaging analysis of non-native .stl files for guided protocols
Source: BMC Oral Health. 2023 Jul 17;23:494. doi: 10.1186/s12903-023-03113-9 (PMC10353103; doi:10.1186/s12903-023-03113-9)
Supplement: Supplementary file 2 — Supplementary Material 2 [file 12903_2023_3113_MOESM2_ESM.docx]

**Supplementary Figure 1.** Iconographic representation of the digital work-flow used for planning miniscrews insertion using Dolphin software and non-native .stl files. A) coronal view of bicortical anchorage planned with customized miniscrew; B) sagittal view of bicortical anchorage planned with customized miniscrew; C) 3D view; D) surgical guide designed.
